# Identification of a 6-month-old baby with a combination of WAGR and Potocki-Shaffer contiguous deletion syndromes by SNP array testing

**DOI:** 10.1186/s41065-020-00132-2

**Published:** 2020-05-23

**Authors:** Yan Meng, Jun Yang, Chan Tian, Jie Qiao

**Affiliations:** 1 Center for Reproductive Medicine, Department of Obstetrics and Gynecology, Peking University Third Hospital, Beijing, 100191 China; 2 National Clinical Research Center for Obstetrics and Gynecology, Beijing, 100191 China; 3 Key Laboratory of Assisted Reproduction (Peking University), Ministry of Education, Beijing, 100191 China; 4 Beijing Key Laboratory of Reproductive Endocrinology and Assisted Reproductive Technology, Beijing, 100191 China; 5 Beijing Advanced Innovation Center for Genomic, Beijing, 100871 China; 6 Peking-Tsinghua Center for Life Sciences, Peking University, Beijing, 100871 China

**Keywords:** WAGR syndrome, Potocki–Schaffer syndrome, Combined deletion syndrome, 11p15.1p11.2 deletion, Oligohydramnios

## Abstract

WAGR 11p13 deletion syndrome is associated with abnormalities including (W) ilms tumor, (A) niridia, (G) enitourinary abnormalities, and growth and mental (R) etardation (WAGR). Potocki–Schaffer syndrome is a contiguous gene syndrome associated with deletions in 11p11.2, principal features of which are multiple exostoses, parietal foramina development delay, mental retardation, and facial dysmorphism. In some cases, males may have enlarged anterior fontanels and genital abnormalities. Each of these syndromes is very rare. Here we report a patient with both WAGR and Potocki–Shaffer syndromes who presented with aniridia, nystagmus, macular dysplasia, enlarged anterior fontanel, mental retardation, ptosis, low-set ears, micrognathia, and atrial septal defect at 6 months old. SNP array revealed a large (26.25 Mb)deletion: arr[hg19]11p15.1p11.2(18742043–44991839)× 1. Genetic testing allowed for diagnosis of this patient at a very young age. In addition to the postnatal phenotype of the patient, we found one prenatal symptom of these syndromes is oligohydramnios, which when present might indicate advanced prenatal diagnosis. This made the possibility of prenatal diagnosis for these syndromes.

## Introduction

WAGR syndrome (OMIM #194072), first described by Miller in 1964 [1], is a rare microdeletion syndrome that predisposes children to the develop Wilms’ tumor (nephroblastoma), aniridia, genitourinary anomalies, and intellectual disability (mental retardation). The prevalence of WAGR syndrome ranges from 1 in 500,000 to 1 million. The cytogenetic basis of WAGR syndrome, a deletion of 11p13, was recognized by Riccardi et al. in 1978 [2]. The deletion fragment (GRCh37: 31806339–32457087 650.75 kb) varies greatly from 1 to 26.5 Mb (https://decision.sanger.ac.uk/syndrome/35 × overview), and always includes four OMIM genes: *PAX6*, *RCN1*, *WT1*, and *WT1-A*. Mutations in *PAX6* (OMIM: 607108) and *WT1* (OMIM: 607102) genes were confirmed to cause developmental disorders. For example, mutations in *PAX6* may cause keratitis hereditary (OMIM:148190), coloboma of optic nerve (OMIM:120430), Peters anomaly (OMIM: 604229), foveal hypoplasia (OMIM:136520), bilateral optic nerve hypoplasia (OMIM: 165550), aniridia (OMIM:106210), or aniridia cerebellar ataxia and mental deficiency (OMIM: 206700), and *PAX6* also has a haploinsufficient effect; mutations in *WT1* can cause Denys-Drash Syndrome (OMIM: 194080), Frasier syndrome (OMIM:136680), nephrotic syndrome, type 4 (OMIM: 256370), Wilms tumor, type 1 (OMIM: 194070), Meacham syndrome (OMIM: 608978), and mesothelioma, somatic (OMIM: 156240). The eponymous Potocki–Shaffer syndrome (PSS, OMIM: 601224), a rare microdeletion syndrome caused by haploinsufficiency of genes located on 11p11.2p12(GRCh37:43994800–46052450, 2.06 Mb), was described for the first time in 1996 [3]. The main phenotypes of PSS include developmental delay, craniofacial abnormalities, mental retardation, multiple exostoses, parietal foramina, enlarged anterior fontanel, ophthalmologic anomalies, and genital abnormalities in males. PSS is inherited in an autosomal dominant manner, and the size of the deletion segment is variable with a minimum content of 2.06 Mb (GRCh37: 31806339–32457087, 2.06 Mb)(https://decipher.sanger.ac.uk/syndrome/34#overview). This area includes 14 OMIM genes. Among them, *EXT2* (OMIM: 608210) is responsible for multiple exostoses (OMIM:133701) [4], and *ALX4* (OMIM: 605420) causes parietal foramina (OMIM: 609597) [5]. Here we document the diagnosis of a female infant suffering from WAGR and PSS concurrently; her heterozygous deletion includes the core genes and she displays a partial phenotype of each syndrome. At 5 months, she was diagnosed as the youngest case reported so far. We also summarize her prenatal stage phenotype to provide crucial indications for prenatal testing and diagnosis for other possible patients.

## Case description

A 4-month-old female infant was referred to the genetic clinic for aniridia and an enlarged anterior fontanel during November 2019. She was the first child of non-consanguineous parents, both of whom are now 33 years old. The mother underwent surgical tumor removal due to mucinous cystadenoma of the right ovary at the age of 27. Simultaneously, she suffered from polycystic ovarian syndrome (PCOS) and subclinical hypothyroidism. Because of the PCOS, the infant was conceived through in vitro fertilization and embryo transfer (IVF-ET). Euthyrox was given orally before pregnancy, and thyroid function was well-controlled. A genetic karyotype analysis of both parents was done prior to in vitro fertilization (IVF), and results were normal.

**Fig. 1:**
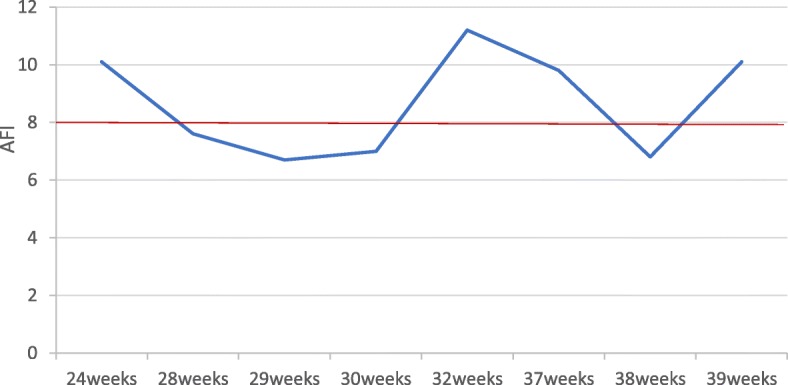
The amniotic fluid index (AFI) in the mid-late stage of gestation. The amount of amniotic fluid in the mid-late stage of gestation. The mother’s amniotic fluid was reduced beginning from the 28th week of gestation. (AFI > 8 is normal, AFI between 5 and 8 is oligohydramnios)

The mother of the patient had a routine prenatal examination during pregnancy. The ultrasound examination at 12 weeks of gestation revealed no major structural abnormalities in the fetus, and the thickness of nuchal translucency (NT) was normal. Noninvasive prenatal testing (NIPT) at 14 ^+ 5^ weeks showed no abnormalities. There was a mild reduction in the amount of amniotic fluid (AF) observed at 28 weeks of gestation, but no other abnormal findings were identified at this time. Percutaneous umbilical blood sampling was recommended to the mother to exclude possible genetic disorders, but she declined. The amount of AF was monitored regularly during mid-late gestation (weeks 24 through 39), and results are shown in Fig. 1. The amount of AF was reduced than normal between 28 and 30 weeks and at 38 weeks of gestation. The baby was born at 40 weeks of gestation. Her birth weight was 2820 g, height was 49 cm, head circumference (HC) was 31.5 cm, and Apgar score was 10; all parameters were in normal range.

After delivery, however, due to “poor response”, the infant was transferred to the neonatal intensive care unit. After a routine blood test showed that the white blood cells (WBC) increased to 33.7 × 10^9^/L and granulocytes were at 72.8%, antibiotic treatment was given. Physical examination showed that the anterior fontanelle was 2.5 × 2.5 cm, full, and accessible to the bone seam, and the posterior fontanelle was at 2x2cm and not closed; thyroid function appeared to be normal; ultrasound scan showed normal liver, gallbladder, pancreas, spleen and kidney; normal MRI brain scan and diffusion-weighted brain imaging was reported; echocardiography showed that the atrial septal defect (central type) was 2 mm; and the newborn hearing screening was in the normal range. After 12 days of antibiotic treatment, the WBC decreased to 9.1 × 10^9^/L, and the granulocytes decreased to 34.6%. The infant was then discharged from the hospital.

Signs of bilateral aniridia and ptosis were noticed by physicians at Xi’an Angel Women’s & Children’s Hospital when the infant was 6 weeks old. Developmental delays were observed at 3 months old with manifestations of great motor retardation. At 4 months old, she was diagnosed with aniridia, ptosis, macular dysplasia, nystagmus, low set ears, and an enlarged anterior fontanel. Genetic examination was recommended. On November 6, 2019, SNP array analysis was performed in our hospital, and arr [hg19]11p15.1p11.2(18742043–44,991,839) xl (26.25 Mb) was detected. The molecular diagnosis confirmed that the patient had both WAGR and Potocki-Schaffer syndromes.

The patient is now 6 months old with normal body length, weight, and head circumference. After intensive pediatric physical therapy, she can raise her head but cannot sit. Her intellectual ability is equivalent to that of a 3-month-old. Physical examination revealed an enlarged anterior fontanel, aniridia, ptosis, macular dysplasia, nystagmus, low set ears, rough face, micrognathia, and atrial septal defect. The growth stage-based development and phenotype assessments are shown in Table 1.

**Table 1:** Growth, development and phenotype of the patient

Age	Weight (kg)	Height (cm)	HC (cm)	Parietal foramina	Abnormality of ophthalmologic	Ear	Face	Development
At birth	2.85	49.0	31.5	Anterior fontanelle 2.5 × 2.5 cm Posterior fontanelle 2.0 × 2.0 cm Accessible to the bone seam	Aniridia Ptosis	Low-set ears	Gothic arch	
3 months	5.40	56.4	37.0	Anterior fontanelle >5x5cm	Aniridia Ptosis	Same as up	Same as up	Developmental delay High tension of limb muscles
4 months	6.40	59.2	38.0	Anterior fontanelle >5x5cm	Aniridia Ptosis Nystagmus	Same as up	Same as up	Developmental delay High tension of limb muscles High back flexion tension
5 months	6.90	61.6	39.4	Anterior fontanelle >5x5cm	Aniridia Ptosis Nystagmus	Same as up	Gothic arch Micrognathia	Developmental delay Limited abduction of both legs Great motor retardation
6 month	7.30	63.1	41.0	Anterior fontanelle >5x5cm	Aniridia Ptosis Nystagmus	Same as up	Same as up	Developmental delay Great motor retardation

## Discussion

We present a patient who was diagnosed by genetic testing with both WAGR and Potocki-Schaffer syndromes at 5 months; to date she is the youngest diagnosis of such a combination. She presented phenotypes including intellectual disability, aniridia, nystagmus, low-set ears, ptosis, macular dysplasia, rough face, micrognathia, atrial septal defect, developmental delay, enlarged anterior fontanel, and atrial septal defect. The only perinatal indication was decreased amniotic fluid.

WAGR syndrome is defined as a genetic syndrome in which there is a predisposition to aniridia, Wilms tumor, genitourinary anomalies, and intellectual disability. Some individuals may also present with mental disorders and obesity. A combination of two or more of the aforementioned clinical features is required for an individual to be diagnosed with WAGR syndrome. The feature invariably present in all documented cases is aniridia [6]. Aniridia can be found in infancy and is often accompanied by late cataracts, glaucoma, and corneal abnormalities. Wilms tumor is usually bilateral and occurs early, and renal dysplasia is also common. Children with WAGR syndrome should receive regular (3–4 monthly) renal surveillance for Wilms tumor until at least the age of 6–8 years and thereafter should remain under some renal follow-up due to the risk of late onset nephropathy (experienced by 40% of patients over the age of 12 years). Genitourinary anomalies include cryptorchidism, hypospadias, and ambiguous sexual organs. Females with WAGR syndrome may have streak ovaries, which can increase their risk for gonadoblastoma, and malformations of the vagina and/or uterus may also be present (https://decipher.sanger.ac.uk/syndrome/35#overview). Given the myriad complications and risks, we will continue to monitor this patient.

WAGR and Potocki–Schaffer syndromes are caused by deletions of genes located on chromosome 11. By searching in DECIPHER (https://decipher.sanger.ac.uk), we confirmed that our patient has the second largest p13 deletion on chromosome 11 after a case with a 134.05 Mb mosaic deletion (DECIPHER ID: 286222). Ten other cases with shorter deletions are (by DECIPHER ID number): 392823(20.55 Mb), 394119(20.55 Mb), 394138(20.55 Mb), 394162(17.80 Mb), 286003(15.14 Mb), 290351(15.55 Mb), 394905(17.80 Mb), 394959(20.55 Mb), 394991(17.80 Mb) and 395910(22.70 Mb). The phenotypic variability among these cases is high. Some patients already exhibit the phenotypes caused by the deletion of key pathogenic genes (*ALX4*, *EXT2*, *PAX6*, and *WT1*), and others do not. Deleted fragments containing the *BNDF*, *E2F8* and *KCNA4* genes found in the current case were not detected in previously reported cases. Therefore, unique phenotypes observed in our patient may appear in other patients in the future. The details of previously reported cases are shown in Supplementary Table 2.

In addition, two similar cases and an atypical case were reported in the literature that are not in DECIPHER (Table 2). Case 1, reported in 1995, was a 26-year-old man with WAGR syndrome and multiple exostoses. Chromosome analysis showed an abnormal male karyotype 46,XY,del(11)(p11.2p14.2) with an interstitial deletion in the short arm of chromosome 11. A deletion of the *WT1* gene was confirmed using FISH probes [7]. Case 2, reported in 2005, was a 25-year-old woman with WAGR and Potocki–Shaffer syndromes. Conventional high-resolution R-banding found a deletion in the proximal short arm of chromosome 11 homologue with breakpoints described as del(11)(p11.2p14.1); the deletion was then confirmed by FISH [8]. Due to the limitations of genetic technology when these cases were discovered, the exact chromosome location of the deleted segments were not available, so we were unable to analyze in detail the associations between the deleted genes and phenotypes. Case 3, reported in 2009, was a 15-year-old boy with an 11p microdeletion including *WT1* but not *PAX6*. Cytogenetic analysis showed a deletion on 11p that was further characterized using FISH and MLPA analyses. The deletion (11p13-p12), located between the deletions associated with WAGR and Potocki-Shaffer syndromes, had a maximum size of 8.5 Mb and encompasses 44 genes. The distal and proximal breakpoints were mapped [9]. While the exact chromosome location of the deletion was obtained in this case, the diagnosis methods used (karyotype analysis, FISH, and MLPA) are tedious and time-consuming.

**Table 2:** Summary of similar cases reported in the literature

Case number	Deletion and the breakpoints	Age at last clinical assessment	Phenotypes	References
Case 1	del(11)(p11.2p14.2)	26 years	Bilateral aniridia, Lacunae in the left parietal, Large fontanelle, Bilateral buphthalmos, Glaucoma, Lens opacities, Hypospadias, Undescended testes, Small penis at 2 months; Wilms’tumour at 2 years; Glomerulonephritis, Multiple exostoses, Hypertension, Mental retardation at 4 years and older.	[7]
Case 2	del(11)(p11.2p14.1)	25 years	Aniridia, Ptosis, Low set ears, Flat malar areas, Micrognathia at 3 months; Left kidney tumor at 15 months; Multiple exostoses at 6 years; Cataract at 7 years; Mammary hypertrophy, Severe obesity at 10 years; Horizontal nystagmus, Bilateral aphakia, Complete bilateral aniridia with neovascularization, Scars of corneal ulcer, Corneal opacities, Ocular hypertension, Hypertension, Proteinuria, Mild to moderate mental retardation, Disturbances, Obsessive, Hyperphagia, Temper tantrums, Intolerance to frustration, Exostoses, Small foramina.	[8]
Case 3 (atypia no *PAX6 *deletion)	del(11)p13-p12	15 years	Cataract, Astigmatism and myopia in the right eye, Facial deformities, Bilateral ptosis, Nasal bridge depression, Ear fold, Maxillary malocclusion, Cryptorchidism, Hypospadias, Postoperative testicular atrophy, Mild to moderate mental retardation, Epilepsy starts at the age of 9.	[9]

For our patient, the deletion size of 11p is 26.25 Mb and includes 138 genes, 73 of which are OMIM genes. *ALX4*, *EXT2*, *PAX6* and *WT1*, all of which affect development, are the key genes for the pathogenesis of WAGR and Potocki–Schaffer syndromes. Other altered genes in this patient that may cause diseases due to haploinsufficiency include *BDNF, CAPRIN1, KCNA4, LGR4*, and *SLC1A2*. Recently, Xu et al. reported that deletion of *SLC1A2, PRRG4*, and *BDNF* may be involved in abnormal psychological development [10] (e.g., early epileptic encephalopathy). Andrey V. et al. reported that deletion of the *PRRG4* gene is related to autism in patients with WAGR, and deletion of both *WT1* and *LMO2* genes is increases the risk of developing Wilms tumor [11]. The phenotypes for *KCNA4* deficiency include microcephaly, cataracts, impaired intellectual development, and dystonia with abnormal striatum (OMIM: 618214). An insufficient dose of *LGR4* can lead to low bone mineral density in adults (OMIM: 615311). In the deletion area, some genes were morbid and can cause the phenotype, and some genes were not. We described some genes that can cause phenotype and other genes were summarized in in Supplementary Table 1. The patient was diagnosed at 5 months old. Her current phenotypes are intellectual disability, aniridia, nystagmus, low-set ears, ptosis, macular dysplasia, rough face, micrognathia, atrial septal defect, developmental delay, enlarged anterior fontanel, and atrial septal defect. According to the previously reported phenotypes of these syndromes and the functions of the associated deleted genes, we anticipate that some phenotypes, such as glaucoma, cataract, Wilms tumor, multiple exostoses, strabismus, autism, dystonia with abnormal striatum, early epileptic encephalopathy, or other mental problems, may occur later in her life. A depressed nasal bridge, streak ovaries, malformations of the uterus, diabetes, and low bone mineral density in adulthood might also appear in the future. Therefore, we suggest follow-ups to monitor the patient for these potential phenotypes as early as possible so that she can receive appropriate treatments in a timely manner. Our report describes a unique case that will help doctors enhance their understanding of this disease.

To improve the perinatal identification of these extremely rare genetic disorders, we searched the literature for prenatal diagnosis indications. We found that only one article reported these characteristics for WAGR syndrome during the prenatal stage [12]. The mother was diagnosed with gestational diabetes at 16 weeks of gestation. The ultrasonographic examination at 29 weeks of gestation found borderline bilateral ventriculomegaly, absent corpus callosum, absent cavum septum pellucidum, large kidneys, and mildly reduced amniotic fluid volume. For further diagnosis, she opted for fetal brain MRI and amniocentesis followed by microarray analysis, and WAGR syndrome was diagnosed before the late termination of pregnancy. Similarly, in our currently reported case, we detected mildly reduced amniotic fluid. Genetic consultation was provided and amniocentesis was recommended. Unfortunately, the mother declined to undergo amniocentesis and we missed the opportunity for prenatal diagnosis. No abnormalities were indicated by other prenatal tests. Oligohydramnios is the common prenatal feature and an indication of these syndromes in both of these cases. It is very important that women with reduced amniotic fluid undergo prenatal diagnosis after prenatal screening. Doctors should continue to collect and report data during the prenatal stage to provide clues for early diagnosis.

## Supplementary information

**Additional file 1: Table S1.** The genes that don’t affect development but cause diseases due to haploinsufficiency.

**Additional file 2: Table S2.** Cases including core genes of two syndromes.

## Data Availability

All data generated or analyzed during this study are included in this published article.

## Abbreviations

OMIM: Online Mendelian Inheritance in Man
IVF-ET: In Vitro Fertilization and Embryo Transfer (IVF-ET)
PCOS: Polycystic Ovary Syndrome (PCOS)
NIPT: Noninvasive Prenatal Testing (NIPT)
NT: Nuchal Translucency (NT)
AF: Amniotic Fluid (AF)
HC: Head Circumference (HC)
AFI: Amniotic Fluid Index (AFI)
WBC: White Blood Cells (WBC)
