# Supplementary material for: Identification of a 6-month-old baby with a combination of WAGR and Potocki-Shaffer contiguous deletion syndromes by SNP array testing
Source: Hereditas. 2020 May 23;157:23. doi: 10.1186/s41065-020-00132-2 (PMC7245943; doi:10.1186/s41065-020-00132-2)
Supplement: Supplementary file 1 — Additional file 1: Table S1. The genes that don’t affect development but cause diseases due to haploinsufficiency. [file 41065_2020_132_MOESM1_ESM.docx]

**Additional file 1 Table 1 The genes that don’t affect development but cause diseases due to haploinsufficiency**

| **Gene** | **OMIM**  **Number** | **pLI** | **Function** |
| --- | --- | --- | --- |
| *CAPRIN1*  cell cycle-associated protein 1 | 601178 | 1.00 | May regulate the transport and translation of mRNAs of proteins involved in synaptic plasticity in neurons and cell proliferation and migration in multiple cell types. |
| *CSTF3*  cleavage stimulation factor | 600367 | 1.00 | One of the multiple factors required for polyadenylation and 3'-end cleavage of mammalian pre-mRNAs. |
| *EIF3M* eukaryotic translation initiation factor 3, subunit M | 609641 | 0.98 | It is required for several steps in the initiation of protein synthesis. The eIF-3 complex specifically targets and initiates translation of a subset of mRNAs involved in cell proliferation. (Microbial infection) May favor virus entry in case of infection with herpes simplex virus.entry-defective, replication-competent porcine kidney cells transferred |
| *EHF*  ets homologous factor | 605439 | 0.98 | Transcriptional activator that may play a role in regulating epithelial cell differentiation and proliferation. May contribute to development and carcinogenesis by acting as a tumor suppressor gene or anti-oncogene. |
| *FBXO3*  f-box only protein 3 | 609089 | 0.97 | Mediates the ubiquitination of *HIPK2* and probably that of *EP300*, leading to rapid degradation by the proteasome. |
| *LRRC4C*  leucine-rich repeat-containing protein 4c | 608817 | 0.95 | May promote neurite outgrowth of developing thalamic neurons. |
| *MPPED2*  metallophosphoesterase domain-containing protein 2 | 600911 | 0.97 | Displays low metallophosphoesterase activity (in vitro). May play a role in the development of the nervous system. |
| *TRAF6*  tnf receptor-associated factor 6 | 602355 | 1.00 | May be essential for the formation of functional osteoclasts. Seems to also play a role in dendritic cells (DCs) maturation and/or activation. |
| *TTC17*  tetratricopeptide repeat domain 17 |  | 1.00 | Plays a role in primary ciliogenesis by modulating actin polymerization. |
| *E2F8*  e2f transcription factor 8 | 612047 | 1.00 | Atypical E2F transcription factor that participates in various processes such as angiogenesis and polyploidization of specialized cells. Plays a key role in polyploidization of cells in placenta and liver. Required for placental development by promoting polyploidization of trophoblast giant cells. |
| *NAV2*  neuron navigator 2 | 607026 | 1.00 | Involved in neuronal development, specifically in the development of different sensory organs. |
