# Supplementary material for: Identification of a 6-month-old baby with a combination of WAGR and Potocki-Shaffer contiguous deletion syndromes by SNP array testing
Source: Hereditas. 2020 May 23;157:23. doi: 10.1186/s41065-020-00132-2 (PMC7245943; doi:10.1186/s41065-020-00132-2)
Supplement: Supplementary file 2 — Additional file 2: Table S2. Cases including core genes of two syndromes. [file 41065_2020_132_MOESM2_ESM.docx]

**Additional file 2 Table 2 Cases including core genes of two syndromes**

| **Decipher ID** | **Age**  **at last clinical assessment** | **Deletion variant** | **Size** | **Sex** | **Phenotypes** | **Lacking haploinsufficient genes compared to our case** | **More haploinsufficient genes than our case** |
| --- | --- | --- | --- | --- | --- | --- | --- |
| 392823 | <1 year | 11:31043425- 51594205 | 20.55Mb | 46, XX | Abnormality of the kidney, Abnormality of the lens, Abnormality of the nares, Aniridia, Aplasia of the ovary, Cataract, Conductive hearing impairment, Intellectual disability, Low-set ears, Micrognathia, Nystagmus, Overlapping toe, Sensorineural hearing impairment, Small for gestational age, Streak ovary, Underdeveloped nasal alae | *BNDF*  *E2F8*  *KCNA4*  *LGR4*  *MPPED2*  *NAV2* | *PHF21A*  *AMBRA1*  *AP15*  *ATG13*  *CELF1*  *CKAP5*  *F2*  *PSMC3* |
| 394119 | 3 years | 11:31043425- 51594205 | 20.55Mb | 46, XY | Aniridia, Intellectual disability, Nystagmus, Proportionate short stature, Protruding ear, Renal neoplasm, Single transverse Palmar crease, Triangular face | *BNDF*  *E2F8*  *KCNA4*  *LGR4*  *MPPED2*  *NAV2* | *PHF21A*  *AMBRA1*  *AP15*  *ATG13*  *CELF1*  *CKAP5*  *F2*  *PSMC3* |
| 394138 | <1 year | 11:31043425- 51594205 | 20.55Mb | 46, XX | 2-3 toe syndactyly, Depressed nasal bridge, Intellectual disability, Postnatal macrocephaly, Protruding tongue, Spasticity | *BNDF*  *E2F8*  *KCNA4*  *LGR4*  *MPPED2*  *NAV2* | *PHF21A*  *AMBRA1*  *AP15*  *ATG13*  *CELF1*  *CKAP5*  *F2*  *PSMC3* |
| 394959 | 39 years | 11:31043425- 51594205 | 20.55Mb | 46, XX |  | *BNDF*  *E2F8*  *KCNA4*  *LGR4*  *MPPED2*  *NAV2* | *PHF21A*  *AMBRA1*  *AP15*  *ATG13*  *CELF1*  *CKAP5*  *F2*  *PSMC3* |
| 394162 | 18 years | 11:31043424-48843424 | 17.80Mb | 46, XY | Abnormality of the pinna, Aniridia, Carious teeth, Cataract, Cryptorchidism, Everted lower lip vermilion, High palate, Intellectual disability, Long face, Long philtrum, Microcephaly, Micropenis, Prominent nasal bridge, Proportionate short stature, Ptosis, Strabismus, Thick lower lip vermilion, Thick upper lip vermilion, Truncal obesity | *BNDF*  *E2F8*  *KCNA4*  *LGR4*  *MPPED2*  *NAV2* | *PHF21A*  *AMBRA1*  *AP15*  *ATG13*  *CELF1*  *CKAP5*  *F2*  *PSMC3* |
| 394905 | 3 years | 11:31043424-48843424 | 17.80Mb | 46, XX | Aniridia, Intellectual disability, Nystagmus, Renal neoplasm | *BNDF*  *E2F8*  *KCNA4*  *LGR4*  *MPPED2*  *NAV2* | *PHF21A*  *AMBRA1*  *AP15*  *ATG13*  *CELF1*  *CKAP5*  *F2*  *PSMC3* |
| 394991 | 10 years | 11:31043424-48843424 | 17.80Mb | 46, XX | Aniridia, Cataract, Misalignment of teeth, Renal neoplasm, Scoliosis | *BNDF*  *E2F8*  *KCNA4*  *LGR4*  *MPPED2*  *NAV2* | *PHF21A*  *AMBRA1*  *AP15*  *ATG13*  *CELF1*  *CKAP5*  *F2*  *PSMC3* |
| 290351 | unknown | 11:3128006- 4682015 | 15.55Mb | unknown | Abnormality of the genitourinary system, Aniridia, Intellectual disability | *BNDF*  *E2F8*  *KCNA4*  *LGR4*  *MPPED2*  *NAV2* | *PHF21A*  *AP15* |
| 395910 | 26 years | 11:26143424-4884324 | 22.7Mb | 46, XY | Abnormal immunoglobulin level, Abnormality of the kidney, Aniridia, Broad neck, Broad ribs, Buphthalmos, Cataract, Coarse Facial features, Cryptorchidism, Delayed closure of the anterior fontanelle, Delayed speech and language development, Enlarged kidney, Extra fontanelles, Gait disturbance, Glaucoma, Hematuria, Hypospadias, Intellectual disability, Low-set ears, Micropenis, Obesity, Osteochondroma, Platyspondyly, Posteriorly rotated ears, Proportionate short stature, Recurrent infections, Renal neoplasm, Short neck, Short ribs, Visual impairment | *BNDF*  *E2F8*  *NAV2* | *PHF21A*  *AMBRA1*  *AP15*  *ATG13*  *CELF1*  *CKAP5*  *F2*  *PSMC3* |
| 286003 | <1year | 11:31284214-46421789 | 15.14Mb | 46, XX | Blepharophimosis, Generalized Hypotonia, High pitched voice, Microphthalmia, Redundant neck skin, Split hand | *BNDF*  *E2F8*  *KCNA4*  *LGR4*  *MPPED2*  *NAV2* | *PHF21A*  *AP15*  *AMBRA1* |
